# The Risk Factors Related to Voice Disorder in Teachers: A Systematic Review and Meta-Analysis

**DOI:** 10.3390/ijerph16193675

**Published:** 2019-09-30

**Authors:** Haewon Byeon

**Affiliations:** Department of Speech Language Pathology, School of Public Health, Honam University, 417, Eodeung-daero, Gwangsan-gu, Gwangju 62399, Korea; bhwpuma@naver.com; Tel.: +82-10-7404-6969; Fax.: +82-50-4461-6969

**Keywords:** voice disorders, teachers, meta-analysis, systematic review, occupational environment, risk factors

## Abstract

*Background and Objectives:* Identifying the risk factors of teachers’ voice disorders is very important for preventing voice disorders and the recurrence of them. This meta-study identified risk factors associated with teachers’ voice disorders through systematic review and meta-analysis and provided basic data for preventing them. *Materials and Methods:* This study collected literature on the risk factors of teachers’ voice disorders using six databases (i.e., CINAHL, EBSCO, PUBMED, SCOPUS, Web of Science, and Springer Link). Search was limited to studies published between 1 January 2000 and 15 October 2018, and a total of 16 publications were selected for the analysis of this study. The quality of selected literature was assessed using the “Standard Quality Assessment Criteria for Evaluating Primary Research Papers from a Variety of Fields”. The effect size was analyzed by odds ratio and 95% confidence interval. *Results:* The results of the quality assessment ranged from 20 to 24 points with six strong studies and ten good studies. The meta-analysis showed that gender, upper airway problems, caffeine consumption, speaking loudly, number of classes per week, and resignation experience due to voice problems were the major risk factors of teachers’ voice disorders. On the other hand, age, number of children, drinking, physical activity, smoking, water intake, singing habits, duration of teaching, perception of noise inside the school, number of classes per day, noise assessment inside the classroom, and perception of technology and instruments inside the workplace were not significantly related to voice disorders. *Conclusions:* Longitudinal studies should be conducted in the future to confirm causality between voice disorders and risk factors based on the results of this study.

## 1. Introduction

Maintaining a healthy voice is important for people who use their voices to conduct professional tasks, such as teachers, singers, and telemarketers [1]. It has been reported that many people using voice for their occupations have a higher incidence of a voice disorder than those not using voice for their occupations due to overuse or misuse of their voices [2]. Particularly, teachers were classified as an occupational group that are more likely to have a vocal issue [1]. Previous studies showed that the prevalence of a voice disorder in this group ranged from 10 to 70% [3,4]. Many teachers suffer from various voice issues such as throat discomfort [5], vocal fatigue [5,6], and hoarseness [7,8,9,10]. It has been also reported that, in severe cases, they experience voice disorders such as vocal nodules [11].

The onset of these voice disorders can cause a negative impact not only on an individual’s health but also in a social aspect. Teachers have decreased job performance due to voice issues [12]. When these voice issues persist, they are more likely to experience leave of absence [13] or sick leave [4]. Moreover, more teachers tend to resign more frequently due to vocal issues [14]. Additionally, voice disorders can decrease the social life and the communication ability of teachers [15].

Previous studies evaluating risk factors associated with the prevalence of voice disorders have reported that voice disorders are related to work environment [12,16,17,18,19], overall health [3,18,20,21,22], and psychological factors [2,23] as well as personal factors such as sociodemographic factors [13,20] and voice overuse [16]. Therefore, it is necessary to identify factors causing voice disorders to teachers in various dimensions and manage them to prevent these disorders. However, previous studies examining the risk factors of teachers’ voice disorders (1) were limited to a particular culture (ethnicity), region, or country [4,6,10,13,16,20,22], (2) focused on primary school or secondary school teachers [16,18,19,23], (3) did not consider all important factors such as sociodemographic factors, working environment, vocal symptoms, socioeconomic factors, health factors, lifestyle, and associated diseases [3,13,20,24], and (4) used different definitions for voice disorders, making it hard to interpret the results of these studies. 

Although several review studies [25,26] reported the risk factors associated with a vocal problem, we are not aware of any meta-analysis study. A review study [27] that examined the risk factors of teachers’ vocal problems conducted various tests, including clinicians’ perceptual evaluations and acoustic assessments, but most of these tests were conducted by self-reporting type questionnaires. Therefore, this study evaluated whether these vocal disorder measurement methods tended to change after 2014, when the previous review study [27] was published, through a systematic review.

It was necessary to conduct a systematic review and a meta-analysis summarizing the results of previous studies in order to prove the scientific basis beyond the region and the culture. This study identified risk factors associated with teachers’ voice disorders through systematic review and meta-analysis and provided basic data for preventing them.

## 2. Materials and Methods

### 2.1. Literature Review

This study collected literature on the risk factors of teachers’ voice disorders using six databases (i.e., CINAHL, EBSCO, PUBMED, SCOPUS, Web of Science, and Springer Link). Moreover, grey literature was searched using Google Scholar and Digital Dissertation on Demand (DDOD). Search was limited to studies published between 1 January 2000 and 15 October 2018 using the following keywords. 

#### 2.1.1. Pubmed

((((dysphonia[mh] OR phonation[mh] OR aphonia[mh] OR hoarseness[mh] OR (dysphon*[tiab]OR phonation disorders[tiab] OR phonation disorder[tiab] OR voice disorder[tiab] OR voice disorders[tiab] OR voice loss OR voice handicap OR hoarse*[tiab] OR voice problem[tiab] OR voice problems[tiab] OR voice loss[tiab])) AND (school teacher[mh] OR (teacher[tiab] OR teachers[tiab] OR instructor[tiab] OR instructors[tiab])) NOT cancer[ti]))).

#### 2.1.2. Web of Science

TI = (dysphon* OR phonat* OR aphoni* OR hoarse OR hoarse* OR phonation disorder$ OR voice disorder$ OR voice problem$ OR voice loss)AND TI = (school teacher $ OR teacher$ OR instructor$) NOT TI = cancer.

#### 2.1.3. EBSCO (ASC, CINAHL, Medline)

TI (dysphon* OR phonat* OR aphoni* OR hoarse OR hoarse* OR phonation disorder OR voice disorder# OR voice problem# OR voice loss) AND TI (teacher OR instructor OR school teacher*) NOT TI cancer.

### 2.2 Literature Selection Criteria

There were four criteria for selecting literature: (1) it must be a publication written in English; (2) it must be an observational study, and the full text of it must be available; (3) technical report or technical note were excluded; and (4) case studies and review studies were excluded. 

**Figure 1:**
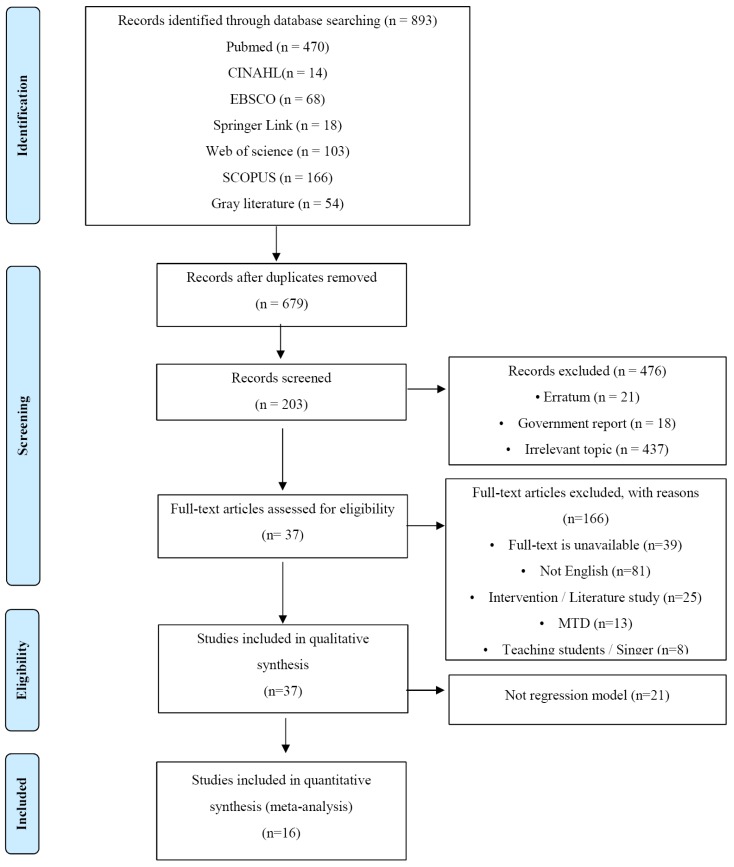
PRISMA flow diagram.

A total of 203 publications were selected in the first round of selection for the analysis topic. Of these, 166 publications were excluded because 81 publications were written in other languages (e.g., Spanish, Chinese, and German), 39 publications were excluded because full-text was not available, 17 intervention publications were excluded, 13 case studies related to muscle tension dysphonia (MTD) were excluded, 8 studies on teaching students and singers were excluded, 8 review studies were excluded, and 21 studies from which it was not possible to identify risk factors using meta-regression analysis due to a continuous variable as a dependent variable (e.g., Jitter) were excluded. Finally, 16 publications were selected for the analysis of this study. This selection process is presented in Figure 1. 

### 2.3 Quality Assessment

The quality of selected literature was assessed using the “Standard Quality Assessment Criteria for Evaluating Primary Research Papers from a Variety of Fields” (SQAC) developed by Kmet et al. (2004) [28]. SQAC is a quality assessment tool that can assess the methodological quality of studies using the criteria for 14 items: objective, study design, subject selection, subject characteristics, random assignment, blinding for study subjects, blinding for researchers, classification bias and assessment methods, sample size, analysis method, an estimate of variance for main results, control of confounding factors, results, and conclusions. Each item was measured by a three-point scale (Yes = 2 points; Partial = 1 point; and No = 0 point). The total score of the assessed items was finally converted to a percentage to indicate the level of a study (strong > 80%; good = 70–80%; adequate = 50–69%; and limited < 50%) [29,30]. The quality of each study was assessed by two researchers independently. When there was a discrepancy between them in the assessment score, a conclusion was drawn by discussing items.

### 2.4 Meta-Analysis

#### 2.4.1. Effect Size Estimation and Interpretation

The effect size was analyzed using the “meta” package of R version 3.5.1 (R Project for Statistical Computing, Vienna, Austria). The values used in the statistical analysis were generated from the number of events in the exposed groups and the unexposed groups. The effect size was analyzed by odds ratio (OR). The significance of effect size was assessed using a 95% confidence interval. 

#### 2.4.2. Homogeneity Test

Homogeneity test was conducted to test the homoscedasticity of the effect sizes derived from individual studies. It was found that the Q statistic (Q-df) of all variables was higher than 0, indicating that there was variance within an individual study and there was high heteroscedasticity between each effect size. Therefore, this study interpreted the results of meta-analysis using a random-effects model.

#### 2.4.3. Publication Bias Test

The publication bias was tested in order to prove the validity of meta-analysis. The results of the funnel plot and the adjusted funnel plot showed that data were scattered near the effect estimates, and there was no publication bias. When trim-and-fill was applied to adjust visual asymmetry, the risk ratio before adjustment (OR = 1.59, 95% CI = 1.27, 2.01) and that after adjustment (OR = 1.38, 95% CI = 1.06, 1.80) were similar. 

## 3. Results

### 3.1. General Characteristics of Literature

Sixteen target studies were classified by year of publication, subject of a study, sampling country, and ethical approval. Five studies were published between 2004 and 2010, and eleven studies were published between 2011 and 2018. Six studies evaluated only elementary school teachers, one study evaluated kindergarten and elementary school teachers, one study evaluated only secondary school teachers, five studies analyzed elementary school and secondary school teachers at the same time, one study targeted kindergarten, elementary, and middle school teachers, one study evaluated municipal school teachers, and one study analyzed teachers and professors at the same time. The number of subjects ranged from 282 to 6039. Sampling countries were Brazil, Nigeria, New Zealand, Lebanon, Malaysia, the United States, Spain, India, Colombia, Poland, South Korea, and Hong Kong. Five studies were conducted in Brazil, while other countries had one study each. Thirteen studies out of 16 studies (81.3%) received ethical approval. 

### 3.2. Results of Quality Assessment

The results of the quality assessment ranged from 20 to 24 points with six strong studies and ten good studies (Table 1). All 16 publications concretely presented the classification bias of objectives, study designs and their results, assessment methods, analysis methods, variance estimation of major results, results, and conclusions. Eight studies randomly assigned samples [3,4,12,13,16,18,19,23], and six studies estimated the sample size in advance (e.g., power analysis) [3,14,19,20,22,24]. All 16 studies controlled confounding variables, but it was not possible to evaluate blinding, mainly used in the intervention method, because they were epidemiological studies.

### 3.3. Definition of Voice Disorders

Voice disorders were defined by using self-reporting questionnaire (*n* = 16), auditory analysis (*n* = 1), acoustic analysis (*n* = 1), and Ear Nose & Throat (ENT) Tests (laryngeal stroboscopy; *n* = 2). All studies basically used self-reporting questionnaires to confirm the risk factors of voice disorders. Survey items included sociodemographic information such as gender, age, and income level [3,4,5,9,10,12,13,16,18,19,20,22,24,31], environment related to work such as classroom environment and the number of students [3,5,9,10,12,13,14,16,18,19,22,24,31], voice habits and voice problem symptoms [3,5,9,10,12,13,14,16,17,19,23,24,31], and health factors such as drug use and infection [12,16,18,19,22]. 

Voice Handicap Index (VHI) [4,14,23] and voice behavior profile [23] were used as objective assessment tools to identify teachers’ voice problems. One study [20] conducted auditory analysis and acoustic analysis using speech samples. Additionally, one study [22] evaluated job satisfaction using Job Stress Scale (JSS), and one study [9] performed a laryngeal stroboscopy test to identify the pathologic laryngeal disease.

### 3.4. Health Level Assessment

Five publications used health-related assessment tools to examine the relationship between health and voice disorder risk factors. Mini-International Neuropsychiatric Interview (MINI) was used to evaluate current depressive episodes [23]. Depression, Anxiety and Stress Scale (DASS-21) was used to test depression, anxiety, and stress [4]. Mental disorders were examined using Self-Reporting Questionnaire (SRQ-20) [22] and General Health Questionnaire-12(GHQ-12) [12], and life quality related to health was evaluated using SF-12/36 [4,24].

### 3.5 Risk Factors of Teachers’ Voice Disorders

The finally selected 16 publications were analyzed to confirm the risk factors of teachers’ voice disorders. The risk factors associated with voice disorders could be divided into sociodemographic factors (e.g., gender, ethnicity, and marital status), health behaviors (e.g., alcohol consumption and smoking), health factors (e.g., depression), voice disorder related diseases (e.g., laryngitis, cold), occupational environment (e.g., noise), and subjective voice problem recognition. 

#### 3.5.1. Sociodemographic Factors and Health Behaviors

Sociodemographic factors related to voice disorders included gender, ethnicity, marital status, and age. Moy et al. (2015) [4] reported that gender was not a risk factor of voice disorders, but other studies [5,13,22,24] showed that women had a higher risk of voice disorders than men. Additionally, singles or divorced/widowed people had a higher risk of voice disorders than married people [4]. Moy et al. (2015) [4] reported that the prevalence of voice disorders was in the descending order of Chinese teachers, Malaysian teachers, and Indian teachers in Malaysia. Moreover, the prevalence of vocal disorders of teachers between 40 and 49 years old was 1.2 times higher than that of teachers in other age groups [4]. In the aspect of age, Moy et al. (2015) [4] showed that teachers between 40 and 49 years old had a 1.2-fold higher risk of suffering from voice disorders than those 50 years old or older. Similarly, Leão et al. [5] showed that teachers 40 years or older had a higher voice disorder risk than those younger than 40 years old, while those between 30 and 39 years old had a lower voice disorder risk than those younger than 30 years old. Moreover, although those at 40 years old and older had a higher risk of voice disorders than those younger than 40 years, those between 30 and 39 years old had a lower risk of voice disorders than those below 30 years old [5]. 

The amount of alcohol consumption, which is one of the health behavior risk factors of voice disorders, proportionally increased the risk of voice disorder onset [3,19]. Moreover, the risk of voice disorders increased when people consumed caffeine more frequently [16] and sang more frequently [31]. However, [16] reported that the risk of voice disorders was low when teachers sang every day. Elementary school teachers sleeping less than six h per day were more likely to have a voice disorder [13], while teachers who did not exercise regularly had a higher risk of voice disorders [12]. Drinking less than four glasses of water increased the development of acute speech disorders [23].

#### 3.5.2. Health Factors

Teachers who experienced depression [23] or anxiety [4] were more likely to experience voice disorders [12] (or teachers who experienced depression [19] or anxiety [4] increased the risk of voice disorder onset than those who did not experience it [12]). Additionally, teachers had the need for rest or sick leave to alleviate the symptoms of voice problems, but the risk of voice disorders increased when teachers could not rest [13,24]. Absence from work due to illness was also a risk factor causing voice disorders [20]. On the other hand, teachers who were not summoned for annual medical check-up [20], who had a higher Short Form Health Survey score [4], and who perceived their own health more positively [24] had a lower risk of voice disorders. 

#### 3.5.3. Diseases Associated with Voice Disorders

Teachers who experienced upper respiratory tract infections such as laryngitis, cold, laryngopharyngitis [3,12,16,18], gastritis [22], thyroid disease [18], and acid reflux [18] had a higher risk of voice disorders.

#### 3.5.4. Occupational Environment

Teachers who held their breathe while speaking in the classroom, clenched their jaw or their teeth while speaking, or experienced high stress had a higher risk or voice disorders [19]. Teachers who had worked for more than 15 years [29] or 20 years [18] had a higher risk of voice disorders than those who had worked for less than 15 years. Moreover, teachers who taught grade four or below had a higher risk of voice disorders than those who taught grade five or above [14]. Furthermore, using a loud voice [12,16,31], excessive voice use [29,31], using voice for an extended period [24], a work issue due to voice disorders [12], and an absence or sick leave due to a voice problem [4,23] were associated with voice disorders. 

In terms of class environment, the risk of teachers’ voice disorder increased when noise generated out of the classroom was louder [19], noise from the surrounding environment during a lecture was louder [3,12,18,19], the classroom was not ventilated properly [12], and the satisfaction of in-classroom teaching aid facilities was lower [22]. Moreover, elementary school teachers had a higher risk of voice disorders than secondary school teachers [5]. Teachers teaching chemical science had a high risk of voice disorders, while those teaching vocational or special education subjects had a lower risk of voice disorders than other teachers [31]. 

#### 3.5.5. Subjective Voice Problem Recognition

The risk of voice disorders was increased when teachers had hyperarousal, voice instability, neck muscle hypertension, hyperfunctional dysphonia [9], hoarseness [10], voice instability, incomplete glottal closure [9], tensed vocalization due to vocal nodules, and voice instability [9]. When teachers experienced vocal fatigue or vocal symptoms over six months, they had a higher risk of voice disorders [10]. Additionally, teachers who recognized voice problems and took medication to improve symptoms [9] or consulted with a language therapist or a doctor had a higher risk of voice disorders [12,19]. On the other hand, the longer the maximum phonation time (MPT) was, the lower the risk of voice disorder was [20].

### 3.6. Meta-Analysis

#### 3.6.1. Relationship between Sociodemographic Factors and Voice Disorders

**Figure 2:**
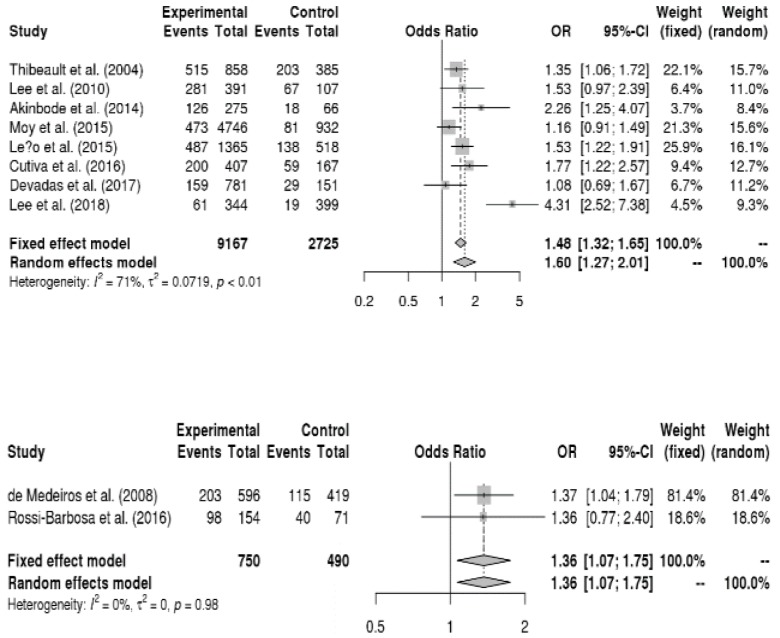
Forest plot about the relationship between vocal disorders and gender (up) and marital status (down).

The relationship between sociodemographic factors and voice disorders was analyzed, and the results are shown in Figure 2. Female teachers had a 1.6-fold higher risk of voice disorders than male teachers (OR = 1.60, 95% CI = 1.27, 2.01). Additionally, married teachers had a 1.4-fold higher risk of voice disorders than single teachers (OR = 1.36, 95% CI = 1.07, 1.75). On the other hand, age and number of children did not significantly affect the onset of a voice disorder. 

#### 3.6.2. Relationship between Disease Factors and Voice Disorders

**Figure 3:**
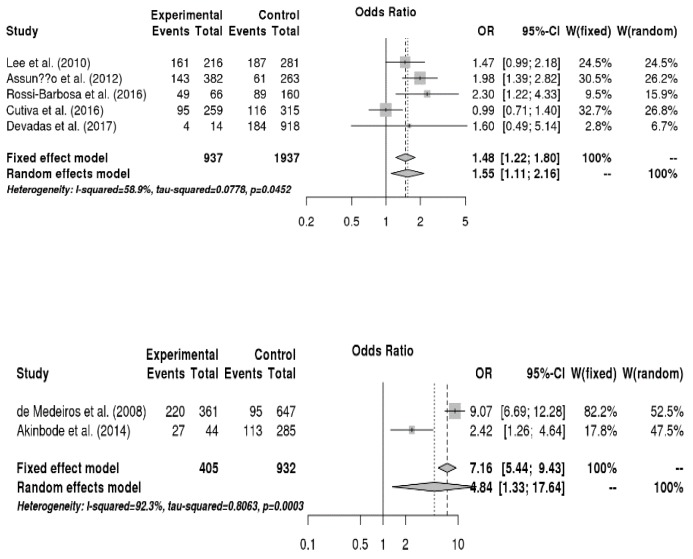
Forest plots regarding the relationship among respiratory allergies, upper respiratory tract infections, and vocal disorders (from up to down in sequence).

The relationship between disease factors and voice disorders was analyzed, and the results are presented in Figure 3. Teachers with respiratory allergies had a 1.6-fold higher risk of voice disorders than those without respiratory allergies (OR = 1.55, 95% CI = 1.11, 2.16). Moreover, teachers who had frequent upper respiratory tract infections had a 4.8-fold higher risk of voice disorders than those who rarely experienced them (OR = 4.84, 95% CI = 1.33, 17.64).

#### 3.6.3. Relationship between Health Risk Behaviors and Voice Disorders

**Figure 4:**
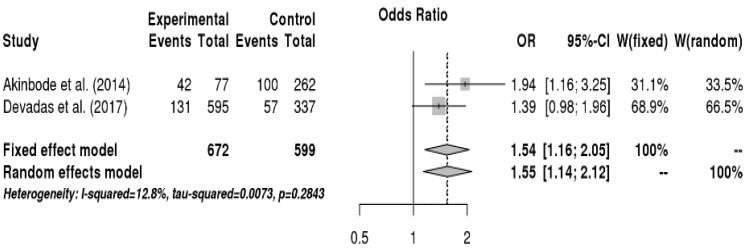
Forest plot about the relationship between caffeine consumption and vocal disorders.

The relationship between health risk behaviors and voice disorders was analyzed, and the results are shown in Figure 4. Teachers who consumed caffeine (e.g., coffee) daily had a 1.6-fold higher risk of voice disorders than those who did not consume or consumed infrequently (OR = 1.55, 95% CI = 1.14, 2.12). On the other hand, drinking, physical activity, smoking, water intake, and singing habits were not significantly related with voice disorders.

#### 3.6.4. Relationship between Occupational Environment and Voice Disorders

**Figure 5:**
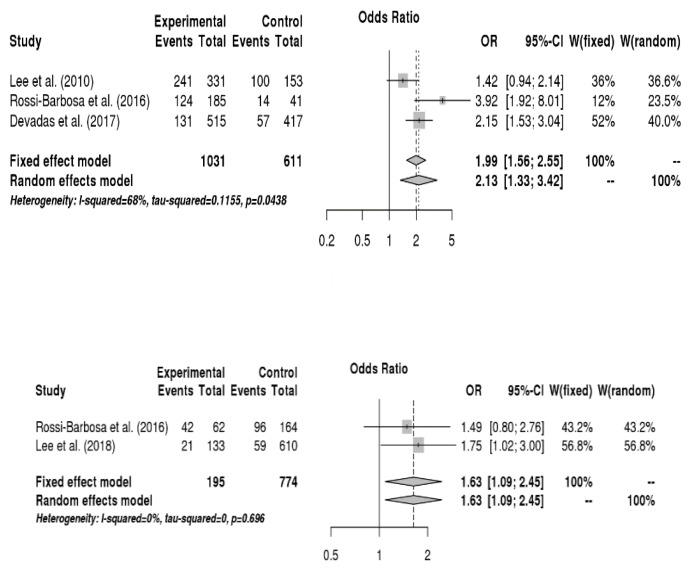

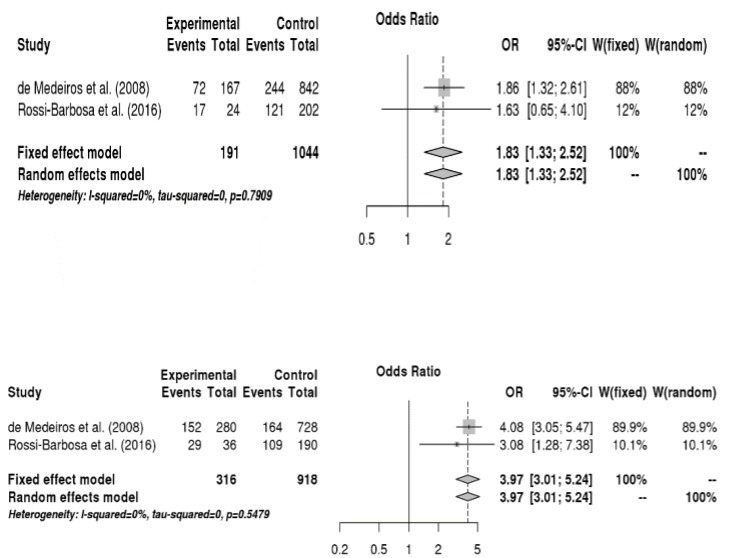
Forest plot showing the relationship between vocal disorders and making a loud voice, the number of classes per week, noise generated out of the school, and leave from work due to voice disorders (from up to down in sequence).

The relationship between occupational environment and voice disorder was analyzed, and the results are shown in Figure 5. Teachers who frequently shouted or yelled had a 2.1-fold higher risk of voice disorders than those who rarely shouted or yelled (OR = 2.13, 95% CI = 1.33, 2.55). Moreover, teachers who gave lectures 20 h or more per week had a 1.6-fold higher chance to have voice disorders than those who taught less than 20 h per week (OR = 1.63, 95% CI = 1.09, 2.45). The risk of voice disorders was 1.8 times higher when the level of noise generated out of the school was unbearable compared to when it was bearable (OR = 1.83, 95% CI = 1.33, 2.52). Additionally, teachers who left from work due to voice disorders had a 4.0-fold higher risk of voice disorders than those who never left from work due to voice disorders (OR = 3.97, 95% CI = 3.01, 5.24). On the other hand, duration of teaching, perception of noise inside the school, number of classes per day, noise assessment inside the classroom, and perception of technology and instruments inside the workplace were not significantly related to voice disorders. 

## 4. Discussion

Identifying the risk factors of teachers’ voice disorders is very important for preventing voice disorders and the recurrence of them. This study systematically evaluated 16 studies published between 2000 and 2018 on the risk factors of teachers’ voice disorders. It was assessed that the quality of these studies were good or better. Nonetheless, only 60% (nine publications) of studies estimated the sample size in advance and sampled subjects [4,5,9,10,12,13,16,20,31]. Future observational studies are needed to estimate the sample size in order to provide high-level grounds. 

Self-reporting questionnaire was the most frequently used assessment tool for measuring a teacher’s voice health among tools defining voice disorders. A review study [26] on teachers’ voice problems conducted 20 years ago showed that almost all studies used self-reporting questionnaires for diagnosing voice disorders. Another review study [27] evaluating studies between 1997 and 2003 revealed that there were qualitative and quantitative changes compared to studies conducted in the 1990s in assessing teachers’ voice disorders by using acoustic and phonetic evaluation tools. The results of this study showed that various measurement methods have been used, such as self-reporting questionnaires [9,32,33,34], aerodynamic assessment [31], acoustic assessment [31,32,35], auditory assessment [31,32,34], video stroboscopy or laryngoscopy [9,32,33], phoniatric examination [9], and noise level assessment [35,36]. Moreover, more studies used ENT tests such as a laryngoscopy test. The diversification of voice problem measures is an important scientific basis for precisely identifying the prevalence and the risk factors of teachers’ voice disorders. Nevertheless, the majority of studies until now have diagnosed voice disorders using different self-reporting questionnaires, and it is a limitation to identify the risk factors of voice disorders. Future studies are required to conduct epidemiological experiments to identify the prevalence of teachers’ voice disorders using standardized assessment tools (e.g., VHI) that can compare it between countries.

The results of this meta-analysis revealed that number of classes per week [13,19], noise generated out of the school [12,19], making a loud voice while lecturing [3,18], and leave from work due to voice disorders [12,19] were risk factors of voice disorders. It is known that lecturing in a noisy environment increases the loudness of voice due to the Lombard effect [37]. When this condition persists, it can eventually lead to laryngeal diseases such as vocal nodules due to overuse and misuse of voice. Classrooms are influenced by the noise generated inside and outside of a school. It is known that the noise generated in classrooms, which includes everyday noise such as students’ chat, fans, and air conditions, is generally between 50–60 dB [38]. On the other hand, the noise generated outside of a school includes the noise from automobiles, construction sites, and airplanes. Therefore, it can go up to 91 dB depending on the environment around a school [39]. Therefore, it is possible that a louder noise from outside of school rather than that from inside of a school may increase the volume of teacher’s voice while teaching to induce vocal disorders to teachers. Moreover, the noise from outside of a school could affect the occurrence of teacher’s vocal disorder more because the noise generated inside of a school can be controlled to some degree (e.g., turning off a fan or an air conditioner or quieting students), but teachers cannot regulate the noise generated outside of a school because it is an external environmental factor.

Moreover, the results of this meta-analysis revealed that teachers who left from work due to dysphonia had four times higher association with voice disorders than those who never left from work due to dysphonia. Voice disorders are known to have a high recurrence rate [40]. Therefore, a teacher who had a dysphonia before is more likely to experience a voice disorder again than a teacher who never had a dysphonia. It may affect the service attitude of the teacher negatively and lead to resignation of the teacher. If a voice problem occurs due to overuse or misuse of voice, teachers will have a hard time to produce a healthy voice, which adversely affects the performance of teachers and ultimately makes them leave from work [12]. It has been reported that voice disorders have a high recurrence rate [41]. Therefore, people with voice disorders are asked to practice efficient vocal hygiene along with resting voice. 

It was also found that diseases related to voice problems were the main factors of teachers’ voice disorders. Neurological diseases, endocrine diseases, and upper respiratory tract diseases were factors causing voice disorders. Particularly, diseases at the upper respiratory tract were found as a major factor causing voice disorders. The overuse and the misuse of a voice and the prevalence of laryngopathy such as laryngitis narrow the airway and cause respiratory diseases [42]. The issues in breathing can adversely affect speech output and cause voice disorders such as vocal nodules and vocal polyps [37]. Consequently, it is necessary to educate teachers on how to eliminate overuse or misuse of a voice, practice proper breathing, and prevent diseases in the upper airway. 

The results of this study showed that female teachers had a 1.6-fold higher risk of voice disorders than male teachers. Although some previous studies [4,16] reported gender did not significantly affect voice disorders, many previous studies argued that gender significantly influenced teachers’ voice disorder [5,13,24]. Moreover, the results of this study agreed with the epidemiological study on the effects of gender on the risk of laryngeal disease [40]. The results implied that, although the voice problems are perceived differently by gender, women might be more vulnerable to voice disorders. 

In terms of health risk behaviors, teachers who consumed caffeine daily were 1.55 times more likely to have a voice disorder than those who did not intake caffeine or had caffeine infrequently. It is not yet fully understood why the group consuming caffeine has a higher risk of laryngeal diseases. One presumable possibility is that beverages containing caffeine can cause gastric acid reflux, which can stimulate the mucosa of the vocal cords to cause a laryngeal disease [43]. However, since there are only a few studies evaluating the relationship between caffeine and voice disorders, future epidemiological studies are needed to evaluate the effects of caffeine on voice disorders in various ethnic and cultural conditions. 

Another finding of this study was that health risk behaviors (e.g., smoking and drinking habits) highly probable to cause voice disorders were not significantly related to voice disorders. Roy et al. (2005) [44] evaluated the association of the last year of smoking, the number of years of smoking, the alcohol consumption more than once a week for more than one year, the first drinking age, and the years of temperance with voice disorders in the U.S. and reported that they were not related to the onset of voice disorders. On the other hand, Byeon & Lee [45] evaluated the relationship between health risk behaviors and laryngeal diseases in South Korea and showed that drinking and smoking were the independent risk factors of laryngeal diseases. The results of this meta-analysis revealed that voice disorders were not significantly related to smoking status and drinking habits (rarely drinking/frequent drinking). The discrepancy could be due to the small sample size (three publications), and it might be limited to draw a robust trend and identify health risk behaviors from them. Moreover, the responses about health behaviors in cross-sectional studies are likely to be affected by recall bias because they depend on subjects’ memories. Long-term follow-up studies are needed to prove the causal relationship between health risk behaviors and voice disorders. Further, it is required to conduct a meta-analysis on a sufficient number of longitudinal studies in order to establish the grounds of future studies. 

The limitations of this study are as follows. First, this study included peer-reviewed publications and grey papers to search for various publications, but it is possible that this study may not have found all publications. Second, this meta-analysis study analyzed papers only published in English and excluded studies published in other languages such as German, Spanish, and French. Third, although this study included various variables to identify the risk factors of voice disorders, studies defined variables differently, and it is possible that there were latent variables (e.g., loudness) that were excluded from the analysis. Therefore, the results should be interpreted carefully. Fourth, this meta-study analyzed only cross-sectional studies, and only 60% of studies estimated the sample size in advance and sampled subjects. Also, there is the possibility of quality assessment tools that are superior to the SQAC used in the study. Therefore, the possibility of low evidence level of this meta-study cannot be excluded. A randomized, controlled meta-analysis with high levels of evidence is needed in the future.

## 5. Conclusions

The results of this meta-analysis showed that gender, upper airway problems, caffeine consumption, speaking loudly, number of classes per week, and resignation experience due to voice problems were the major risk factors of teachers’ voice disorders. Longitudinal studies should be conducted in the future to confirm causality between voice disorders and risk factors based on the results of this study.

## Figures and Tables

**Table 1:** Results of standard quality assessment criteria for evaluating primary research papers from a variety of fields.

Study	Items on Standard Quality Assessment Checklist														
	1	2	3	4	5	6	7	8	9	10	11	12	13	14	Total
Thibeault et al. (2004)	+	+	+	+	-	N/A	N/A	+	±	+	+	+	+	+	21
Sliwinska-Kowalska et al. (2006)	+	+	+	±	-	N/A	N/A	+	±	+	+	+	+	+	20
Hamdan et al. (2007)	+	+	±	+	-	N/A	N/A	+	±	+	+	+	+	+	20
de Medeiros et al. (2008)	+	+	+	+	+	N/A	N/A	+	±	+	+	+	+	+	23
Lee et al. (2010)	+	+	+	+	+	N/A	N/A	+	+	+	+	±	+	+	23
De Alvear et al. (2011)	+	+	+	±	-	N/A	N/A	+	+	+	+	±	+	+	20
Assunção et al. (2012)	+	+	±	+	-	N/A	N/A	+	+	+	+	+	+	+	21
Akinbode et al. (2014)	+	+	+	+	+	N/A	N/A	+	±	+	+	+	+	+	23
Leão et al. (20015)	+	+	+	+	-	N/A	N/A	+	±	+	+	+	+	+	21
da Rocha et al. (2015)	+	+	+	+	+	N/A	N/A	+	±	+	+	±	+	+	22
Moy et al. (2015)	+	+	+	+	+	N/A	N/A	+	±	+	+	+	+	+	23
Cantor Cutiva et al. (2016)	+	+	+	+	-	N/A	N/A	+	+	+	+	+	+	+	22
Rossi-Barbosa et al. (2016)	+	+	+	+	+	N/A	N/A	+	+	+	+	±	+	+	23
Devadas et al. (2017)	+	+	+	+	+	N/A	N/A	+	±	+	+	±	+	+	22
da Rocha et al. (2017)	+	+	+	+	-	N/A	N/A	+	+	+	+	±	+	+	21
Lee et al. (2018)	+	+	+	+	+	N/A	N/A	+	±	+	+	+	+	+	23

+ = yes, ± = partial, - = no, N/A = Not Applicable.
